# Comparison of Physiological Responses between a W´BAL-INT Training Model and a Critical Power Test

**DOI:** 10.5114/jhk/186976

**Published:** 2024-07-17

**Authors:** Miguel Ángel Galán-Rioja, Fernando González-Mohíno, Javier Abián-Vicen, José María Gonzalez-Ravé

**Affiliations:** 1 Sport Training Laboratory, Faculty of Sport Sciences, University of Castilla la Mancha, Toledo, Spain.; 2 Faculty of Health, International University of La Rioja, Logroño, Spain.; 3 Facultad de Ciencias de la Vida y la Naturaleza, Universidad Nebrija, Madrid, Spain.; 4 Performance and Sport Rehabilitation Laboratory, Faculty of Sport Sciences, University of Castilla la Mancha, Toledo, Spain.

**Keywords:** cycling, performance, endurance training, exercise intensity

## Abstract

This study aimed to compare acute physiological responses during the W prime (W´) balance training model (W´BAL-INT) with performance in the critical power test (CP_Test_). Additionally, the study sought to determine the extent of neuromuscular and metabolic fatigue associated with severe and extreme intensity domains. Fourteen road master cyclists (13 male, 1 female) completed graded incremental exercise tests to determine their maximum oxygen uptake and 12−, 7− and 3-min maximal efforts to assess CP and W´ (CP_Test_). Additionally, they participated in a reconstitutive intermittent training session following the W´BAL-INT model. Physiological responses including oxygen uptake (V˙O_2_), the heart rate (HR), blood lactate (BLa̅) concentration, and perceptual responses (RPE), were measured and compared to CP_Test_ performance data. The W´BAL-INT induced steady-state physiological responses in V˙O_2mean_ (F = 0.76, p = 0.655) and absolute HR, relative HR and HR_CP_ (F = 0.70, p = 0.704; F = 1.11, p = 0.359; F = 1.70, p = 0.095, respectively) comparable to CP_Test_. During the 3-min work intervals in the training session, V˙O_2_ was stable and similar to V˙O_2peak_ (54.2 ± 6.7 to 59.3 ± 4.9 ml·kg^−1^·min^−1^) in the CP_Test_. Furthermore, 4-min rest intervals facilitated recovery up to moderate fatigue levels (80–100% of W´ balance). HR responses were sensitive to interval intensity and accumulated time. Meanwhile, BLa̅ responses and the RPE increased fatigue development during W´BAL-INT. The W´BAL-INT training model generates consistent physiological responses in mean oxygen kinetics, the percentage of CP and the HR, similar to those observed during the CP_Test_. However, different physiological responses were observed in peak oxygen kinetics and W´ energy balance.

## Introduction

Training intensity is habitually prescribed using intensity domains (moderate, heavy, severe, and extreme) (Burnley and Jones, 2007). These domains are delimited by physiological thresholds. For example, the transition between the moderate and heavy domains, as well as between the heavy and severe domains can be determined by the gas exchange threshold (GET) and critical power (CP), respectively (Poole et al., 2021; Poole and Jones, 2012). Indeed, prescribing training intensity based on physiological thresholds (particularly CP) can reduce variability in exercise tolerance and acute metabolic responses (Meyler et al., 2023; Poole and Jones, 2023). Moreover, it can be prescribed in a more consistent and effective way in relation to CP than traditional variables such as maximum oxygen uptake (V˙O_2max_) (Collins et al., 2022).

The curvilinear relationship between power output and time, and thus the asymptote (CP) along with the curvature of the power-time relationship representing the work capacity above CP (W´) define the tolerance to continuous and intermittent exercise (Monod and Scherrer, 1965). Therefore, CP may be regarded as a ‘fatigue threshold’ that determines neuromuscular and metabolic fatigue. Since physiological responses to exercise can be stabilized below this threshold but not above it, it allows for the separation of central and peripheral fatigue (Poole et al., 2016; Zoladz et al., 2020). In this way, CP represents the maximal metabolic steady state (MMSS) (Galán-Rioja et al., 2020; Jones et al., 2019), while W´ represents the additional non-oxidative energy capacity expressed in joules (J) or kilojoules (kj) during exercise above CP (Poole et al., 2016). The application of this concept provides an essential foundation for understanding the development of fatigue during exercise across different intensity domains (Black et al., 2016; Jones and Vanhatalo, 2017; Chamera et al., 2023).

Exercise tolerance is associated with power output production and the duration of the exercise bout, as well as the recovery period within an intermittent session (Morton and Billat, 2004). Depletion of W´ begins when power output exceeds CP, while reconstitution occurs when it falls below CP. In this sense, W´ balance can explain the necessity of increase or decrease power output during moderate exercise during training and competition (Skiba et al., 2012; Skiba and Clarke, 2021), providing relevant information of the development of fatigue during intermittent- and constant-intensity exercise (steady state) (Jones and Vanhatalo, 2017).

Recently, Galán-Rioja et al. (2022) determined the utility of integrating the W´ balance model (W´_BAL-INT_) in designing intermittent training a programme. The four-week training programme consisted of varied interval duration (short, medium and long intervals), determined from physiological demands and the performance profile of road cyclists (Sanders and van Erp, 2021). All interval sessions (SML-INT) were designed to completely deplete W´ as assessed by the W´_BAL-INT_ model. After four weeks of training, CP improved by 5%. In addition, mean maximal power over 12, 7 and 3 min increased significantly in the SML-INT group by 9%, 4% and 5%, respectively. However, changes in physiological responses associated with W´ balance were not measured (oxygen uptake (V˙O_2_), the heart rate (HR), blood lactate [BLa̅] concentration and perceptual responses such as the rate of perceived exertion (RPE), as well as the level of fatigue in the severe and extreme intensity domains).

Bearing this in mind, this study aimed to compare the acute physiological responses during intermittent W´_BAL-INT_ training with the CP_Test_ performance test and determine the levels of neuromuscular and metabolic fatigue associated with severe and extreme intensity domains.

## Methods

### Participants

Fourteen (13 male and 1 female) road master cyclists (mean ± SD age = 31.7 ± 9.0 yrs, body mass = 70.1 ± 8.7 kg, body height = 1.77 ± 0.07 m, V̇O_2peak_ = 57.5 ± 4.5 ml·kg^−1^·min^−1^, critical power [CP] = 3.9 ± 0.3 W·kg^−1^, W´= 14.8 ± 4.8 kJ) completed this study. At first, the study sample included 16 participants, yet two female athletes were excluded due to illness during the investigation. Cyclists had no injury or illness during the three months prior to the study commencement. Cyclists were classified as performance level 3 cyclists (De Pauw et al., 2013), and categorised as Tier 3 (national level) according to the classification framework (McKay et al., 2022). The investigation was approved by the Universidad Nebrija (approval code: UNNE-2020-010; approval date: 10 October 2020) and adhered to the principles of Declaration of Helsinki.

### Experimental Design

Cyclists visited the laboratory in randomized order on three occasions within a period of 5 to 6 days. Each test was conducted on a different day, with a 48- to 72-h recovery interval between tests. All testing took place at the same time of the day and under controlled laboratory conditions (ambient humidity ~40% and temperature ~22°C). For all tests, cyclists used their personal racing bikes, which were mounted to a Cyclus2 ergometer (RBM Electronics, Leipzig, Germany) with a fan installed in front of the participant. Cyclists were instructed to avoid intense exercise, alcoholic beverages, and caffeinated drinks 24 h before each test. They were also advised to consume a light meal 3 h before the tests. Body mass and height of each cyclist were measured using a Seca 700 balance with a stadiometer (Seca 700, Seca ltd, Germany) with cyclists wearing light clothing and no shoes. After collecting anthropometrical data, the incremental test was performed until exhaustion. This test allowed to determine the gas exchange threshold (GET), maximum oxygen uptake (V˙O_2max_), the heart rate (HR), and ratings of perceived exertion (RPEs). All cyclists were familiarized with the 10-point Borg scale CR-10 (Borg, 1998). On the next visit, they performed a time-trial test (TT) to calculate CP and W´ (Karsten et al., 2015, 2018). On the last visit, the W´_BAL-INT_ model training session was performed (Galán-Rioja et al., 2022) to determine physiological responses (V˙O_2_, HR and [BLa̅]), and perceptual responses (RPE) in each of the work and recovery intervals.

### Graded Incremental Exercise Test

During the first visit, cyclists completed a graded incremental exercise test to exhaustion. The test was preceded by a 5-min warm up at 50 W and 75 W (for female and male participants, respectively). The starting power of the incremental test was individualized to 75 W and 100 W (for female and male participants, respectively). The load increased continuously and linearly with 25 W·min^−1^ until volitional exhaustion (Lucia et al., 2000), as this would result in test duration of 8–12 min. Cyclists were allowed to choose their own cadence. When the cadence decreased by more than 10 RPM for more than 10 s, the test was terminated.

Oxygen uptake (V˙O_2_) and carbon dioxide output (V˙CO_2_) were measured using a breath-by-breath gas analyzer (CPX/D Med Graphics, St. Paul, MN, USA). Before each test, the analyzer was calibrated with a known gas mixture (12% O_2_ and 5% CO_2_) and the volume sensor was calibrated with a 3-L syringe. The HR was measured during the test with a HR monitor (H10 Sensor; Polar, Kempele, Finland). The V˙O_2_ plateau during the last 30s was defined to verify the achievement of V˙O_2max_ (Poole and Jones, 2017).

### Determination of Critical Power

On their second visit to the laboratory, cyclists completed the CP test (CP_Test_). The test consisted of performing three TT, preceded by a 5-min warm up at 50 W and 75 W (for female and male participants, respectively). The TT used were 12-, 7- and 3-min maximal efforts, with a 30-min low intensity recovery period in between. Linear regression was used to calculate CP and W′ using the power-1/time (P = W′(1/t) + CP) model (Karsten et al., 2015, 2018). V˙O_2_ and HR data were continuously collected throughout the entire test. Mean values for each maximal effort were used. Cyclists were considered to have achieved their V˙O_2max_, when at least two of the following criteria were met: a plateau in V˙O_2_ defined as an increase of less than 1.5 mL·kg^–1^·min^–1^ in two consecutive workloads; a respiratory-exchange ratio >1.15 and a maximal heart rate above 95% of the age-predicted maximum (220 – age) (Howley et al., 1995). If two of these criteria where not achieved, then V˙O_2peak_ was considered. V˙O_2max/peak_ was defined as the highest 30-s mean value recorded during the test. The RPE (range from one to ten) was obtained immediately after each maximal effort.

### W´ BAL-INT Model Training Session

During the last visit to the laboratory, all cyclists performed a training session, after a 15-min warm-up using power of 50 W and 75 W (for female and male participants, respectively). Each interval consisted of 30 s, 1, 3 and 7 min at extreme or severe intensity, interspersed woth 30 s, 1, 3 and 4 min of active recovery at low intensity (<75 W), respectively (Galán-Rioja et al., 2022). The intermittent W´_BAL-INT_ model was used to design a combination of intervals that would result in the complete depletion of W´ by intervals of short (<2 min), medium (2–4 min) and long (>4 min) duration (Rosenblat et al., 2020). V˙O_2_ and the HR were measured during the whole training session and mean values for each interval were obtained. Then, V˙O_2peak_ was defined as the maximum V˙O_2_ value reached during training that did not meet the V˙O_2max_ criteria. The RPE was obtained immediately after each effort. At the end of each exercise bout, 20μl of blood from the fingertip was collected and analyzed for [BLa̅] concentration (Lactate Scout, SensLab GmbH, Germany).

### Statistical Analysis

Statistical analyses were conducted using GraphPad Prism 9.4.1 for macOS (GraphPad Software, San Diego, CA, USA) with significance set at *p* < 0.05. Additionally, 95% confidence intervals/limits were presented. Descriptive data were reported as mean and standard deviation (SD). Data were screened for normality with the Shapiro-Wilk test, which showed that all data had a normal distribution (*p* > 0.05). Physiological and perceptual variables (i.e., V˙O_2_, CP and W´, W´ balance, GET, HR, [BLa−], RPE, and metabolic equivalents (METs)) were analyzed using one-way ANOVA with Dunnett’s multiple comparisons showing the differences between the training model W´_BAL-INT_ and the CP_Test_. Correct multiple comparisons were performed using Brown-Forsythe testing.

## Results

Descriptive characteristics of participants during the CP_Test_ and the graded incremental exercise test are shown in Table 1.

**Table 1:** Physiological characteristics of master cyclists from graded incremental exercise test and critical power test

Variable	All subjects (n =14)
Age (years)	31.71 ± 9.01
Body height (m)	1.77 ± 0.07
Body mass (kg)	70.12 ± 8.72
BMI (kg·m^−2^)	22.33 ± 1.81
V̇O_2peak_ (ml·kg^−1^·min^−1^)	57.54 ± 4.53
CP (ml·kg^−1^·min^−1^)	46.60 ± 5.21
GET (ml·kg^−1^·min^−1^)	37.42 ± 4.80
W´(kJ)	14.82 ± 4.81
HR_max_ (bpm)	184 ± 9
HR_CP_ (bpm)	170 ± 9
HR_GET_ (bpm)	141 ± 12

Data are mean ± SD. Abbreviations: BMI, body mass index; VO_2max_, maximum oxygen uptake; CP, critical power; W´, work above critical power; GET, gas exchange threshold; HR, heart rate

### Oxygen Uptake Kinetics

Table 2 shows the comparison between the physiological responses obtained during the W´_BAL-INT_ intermittent model and during the CP_Test_. There was no main effect of V˙O_2mean_ (*F* = 0.76, *p* = 0.655), neither V˙O_2peak_ (*F* = 0.50, *p* = 0.869). However, significant differences were observed for V˙O_2mean_ relative to CP and GET (*F* = 2.92, *p* < 0.004; *F* = 4.56, *p* < 0.001, respectively), being lower in CP and higher in GET for W´_BAL-INT_ compared to the CP_Test_. Furthermore, significant differences were detected for V˙O_2peak_ relative to CP and GET (*F* = 3.34, *p* < 0.001; *F* = 3.39, *p* < 0.001, respectively), being lower in CP and higher in GET for W´_BAL-INT_ compared to the CP_Test_.

**Table 2:** Physiological responses of intermittent W´_BAL-INT_ model in comparison to performance CP_Test_

n = 14	Short Intervals (S)				Medium Intervals (M)		Long Intervals (L)		SML-INT	CP_Test_
	W´_BAL-INT_ Work	W´_BAL-INT_ Recovery	W´_BAL-INT_ Work	W´_BAL-INT_ Recovery	W´_BAL-INT_ Work	W´_BAL-INT_ Recovery	W´_BAL-INT_ Work	W´_BAL-INT_ Recovery	W´_BAL-INT_ Work/Recovery	
	30-s	30-s	1-min	1-min	3-min	3-min	7-min	4-min	20-min	
Variable										
V̇O_2mean_ (mL·kg^−1^min^−1^)	32.2 ± 5.2^***^	41.5 ± 4.6	45.5 ± 5.4	40.4 ± 5.2	48.9 ± 6.5	30.6 ± 3.7^***^	46.8 ± 6.4	25.1 ± 3.6^***^	40.5 ± 4.9	46.6 ± 5.2
%CP	70 ± 12^***^	91 ± 12	99 ± 13	88 ± 15	107 ± 15	67 ± 9^***^	102 ± 13	55 ± 9^***^	88 ± 15	100%
%GET	88 ± 19	113 ± 21	124 ± 25^*^	110 ± 23	133 ± 28^***^	83 ± 16	127 ± 25^**^	68 ± 14^***^	110 ± 21	
V̇O_2peak_ (mL·kg^−1·^min^−1^)	42.4 ± 6.1^***^	44.5 ± 5.1^***^	50.2 ± 5.8^**^	49.6 ± 5.4^***^	54.2 ± 6.7	50.9 ± 7.6^**^	52.6 ± 6.5^*^	45.5 ± 6.8^***^	54.5 ± 6.7	59.3 ± 4.9
%CP	72 ± 9^***^	75 ± 7^***^	85 ± 8^***^	84 ± 8^***^	91 ± 8	86 ± 9^***^	89 ± 7^**^	77 ± 10^***^	92 ± 7	100%
%GET	116 ± 25	121 ± 23	137 ± 26^**^	135 ± 26^**^	148 ± 30^***^	139 ± 29^**^	143 ± 29^***^	124 ± 26	149 ± 30^***^	
W' depletion (kJ)	9.5 ± 2.5^***^	0^***^	7.4 ± 1.1^***^	0^***^	6.7 ± 2.1^***^	0^***^	4.6 ± 2.2^***^	0^***^	28.3 ± 5.2^***^	14.8 ± 4.8
%W´ depletion	67 ± 17^***^	0	55 ± 20^***^	0	50 ± 23^***^	0	33 ± 16^**^	0	207 ± 66^***^	0%
W' balance (kJ)	5.8 ± 3.4^***^	6.3 ± 3.5^***^	0.2 ± 4.1^***^	2.5 ± 3.8^***^	1.6 ± 4.3^***^	6.4 ± 4.4^***^	9 ± 4.9^**^	11.7 ± 4.9	11.7 ± 4.9	14.8 ± 4.8
%W´ balance	36 ± 16^***^	40 ± 15^***^	-6 ± 31^***^	11 ± 26^***^	2 ± 33^***^	38 ± 23^***^	58 ± 17^***^	77 ± 12^*^	77 ± 12^*^	100%
%W´ reconstitution	4 ± 1^***^		17 ± 8^***^		35 ± 13^***^		19 ± 7^***^		83 ± 25^**^	
MMP (W)	596 ± 107^***^	92 ± 28^***^	402 ± 59^***^	87 ± 25^***^	311 ± 52	73 ± 17^***^	275 ± 46	61 ± 17^**^	208± 31^**^	274 ± 45
HR (bpm)	136 ± 17^***^	160 ± 13	166 ± 12	159 ± 12	170 ± 11	138 ± 13^***^	164 ± 25	133 ± 11^***^	156 ± 11	170 ± 9
%HR_max_	74 ± 8^***^	87 ± 4	90 ± 3	86 ± 4	92 ± 2	75 ± 4^***^	89 ± 14	72 ± 3^***^	85 ± 2^**^	92 ± 2
%HR_CP_	80 ± 9^***^	94 ± 5	97 ± 3	94 ± 4	100 ± 3	81 ± 5^***^	97 ± 15	78 ± 4^***^	92 ± 3^**^	100%
%HR_GET_	96 ± 11	113 ± 8^**^	117 ± 8^***^	113 ± 9^**^	120 ± 9^***^	98 ± 10	116 ± 18^***^	94 ± 9	111 ± 8^*^	
Blood Lactate [BLa̅]	2.6 ± 1.6^***^	-	9.6 ± 3.4^***^	-	13.6 ± 3.3	-	12.9 ± 3.3	-	-	14.8 ± 4.8
RPEs (1–10)	7 ± 3	-	8 ± 2	-	9 ± 1^*^	-	10^**^	-	-	7 ± 1
METs	9.2 ± 1.5^***^	11.9 ± 1.2^***^	13.1 ± 1.4^***^	11.4 ± 1.6^***^	13.8 ± 1.9^***^	8.6 ± 1.2^***^	13.4 ± 1.8^***^	7.1 ± 1.2^***^	11.7 ± 1.3^***^	16.9 ± 1.4

### W´ Energy Balance Kinetics and Maximal Mean Power

There was a main effect for absolute W´ depletion, relative to W´ depletion, W´ balance and W´ reconstitution (*F* = 12.5, *p* < 0.001; *F* = 20.8, *p* < 0.001; *F* = 3.81, *p* < 0.001; *F* = 7.36, *p* < 0.001, respectively), being lower in absolute W´ depletion, relative W´ balance and W´ reconstitution, and higher in relative to W´ depletion for W´_BAL-INT_ compared to the W´ at CP_Test_. However, no significant difference was detected for absolute W´ balance (*F* = 0.21, *p* = 0.993). In addition, there was a main effect for absolute mean maximal power (MMP) (*F* = 3.40, *p* < 0.001), being higher for W´_BAL-INT_ work and lower for W´_BAL-INT_ recovery compared to the CP_Test_.

### Heart Rate Responses

There was no main effect of absolute HR, relative HR_max_, and HR_CP_ (*F* = 0.70, *p* = 0.704; *F* = 1.11, *p* = 0.359; *F* = 1.70, *p* = 0.095, respectively). However, significant differences were observed for HR relative to GET (*F* = 2.47, *p* < 0.012), being lower in CP and higher in GET for W´_BAL-INT_ compared to CP_Test_.

### Blood Lactate, Metabolic Equivalents and Perceptual Responses

There was no main effect of absolute blood lactate concentration compared to W´ at the CP_Test_ (*F* = 1.86, *p* = 0.128; r^2^ = 0.635, *p* < 0.001). Furthermore, there was no main effect for metabolic equivalents (METs) compared to CP_Test_ (*F* = 0.77, *p* = 0.648). However, there was a main effect for the RPE (*F* = 6.60, *p* < 0.001), being the same or higher for W´_BAL-INT_ compared to CP_Test_.

## Discussion

This study demonstrates the sensitivity of CP and W´ to acute physiological responses, such as V˙O_2_ and the HR, when using the W´_BAL-INT_ model. This sensitivity is crucial for exercise prescription during training, and for determining the levels of neuromuscular and metabolic fatigue associated with severe and extreme intensity domains.

**Figure 1:**
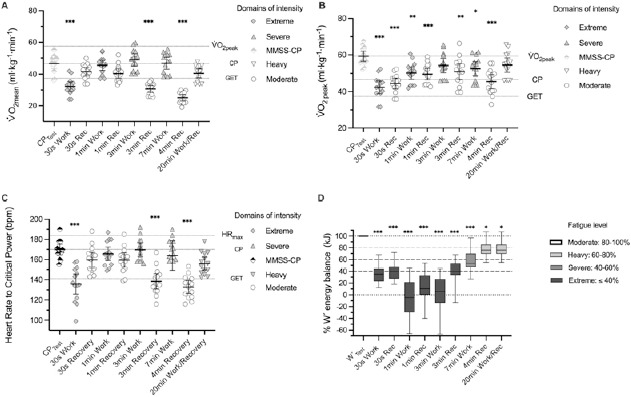
Individual values of the cyclists in each intensity domain of V˙O_2mean_ (A), V˙O _2peak_ (B), Heart rate at Critical Power (C), and energy balance W´ (D) during the Reconstitutive intermittent training model W´ _BAL-INT_. Abbreviations: V˙O_2mean_, mean oxygen uptake; V˙O_2peak_, peak oxygen uptake; W´, finite work capacity above critical power; GET, gas exchange threshold; MMSS, maximal metabolic steady state; CP, critical power; HR, heart rate. Values are presented as mean ± SD. Significantly different at * p < 0.05; ** p < 0.01; *** p < 0.001, alpha level of 0.05 (95% confidence interval).

During the W´_BAL-INT_ training model, the 30-s and 1-min intervals of recovery, as well as the 1-min, 3-min and 7-min intervals of work, accumulated to 12 min and 30 s of the V˙O_2_ steady state compared to V˙O_2mean_ at the CP_test_ (Figure 1A). This is consistent with continuous exercise in the heavy intensity domain, where the slow oxygen component stabilizes after 10–20 min allowing for exercise tolerance (Burnley and Jones, 2007). These acute responses associated with the steady state of V˙O_2_ during the work and recovery times create a suitable oxygen steady state environment. If repeated chronically, this would be expected to promote different physiological adaptations (Holloszy and Coyle, 1984). Additionally, V˙O_2_ of these intervals represents 88–107% of CP, suggesting that prescribing intervals relative to CP may be a better option than relative to V˙O_2max_ (Collins et al., 2022; Meyler et al., 2023), mainly because relating exercise training intensity to CP explains substantially more of the physiological variability and adaptations to training, as well as severe intensity exercise tolerance, than does V˙O_2max_.

Furthermore, during each of the intermittent bouts in the W´_BAL-INT_, the values of V˙O_2mean_ were between GET and CP thresholds (zone 2 in the triphasic model), as indicated by the values relative to GET and CP (Table 2). This reflects the bioenergetic response and fatigue processes associated with exercise intensity domains, kinetics, and the slow component of oxygen (Burnley and Jones, 2018; Gaesser and Poole, 1996).

Regarding V˙O_2peak_, different responses were found in relation to the relative values of CP and GET. However, it was observed that oxygen uptake during the 3-min work interval remained stable and was similar to V˙O_2peak_ during the CP_Test_ (Burnley and Jones, 2018). Importantly, CP represents a limit above which exercise results in the achievement of V˙O_2max_, provided the exercise can be sustained long enough (i.e., ≥ approximately ~2–3 min) to reach it (Hill et al., 2002; Poole et al., 1988; Vanhatalo et al., 2016). This may be due to the mismatch between the neuromuscular energy demand and the instantaneous energy supply, as well as the accumulations of metabolites produced by the previous intervals of severe and moderate intensity without the appearance of the slow component of oxygen (Burnley and Jones, 2018), which depend to a certain extent on the intensity and duration of the exercise. This response may play a role in reaching V˙O_2peak_ during W´_BAL-INT_ and improving performance during this interval as demonstrated in a recent study (Galán-Rioja et al., 2022)

W´ balance was found to be proportional to the interval intensity and the accumulated time during the W´_BAL-INT_ training model, up to the 4-min recovery interval, where W´ balance approached 80%. Therefore, it appears that a 4-min recovery period at the end of the intermittent W´_BAL-INT_ training model is sufficient to regenerate energy up to a moderate fatigue level (80–100% of W´ balance) (Figure 1D). This is consistent with the intermittent exercise tolerance applied to the concept of CP (Chidnok et al., 2012; Morton and Billat, 2004). Additionally, it was observed that the energy (kj) requirements associated with the development of fatigue beyond CP (i.e., W´) differ according to the intensity domain in which the exercise is performed (Black et al., 2016). This highlights the use of CP and W´ to prescribe exercise intensities as a sensitive and variable stimulus (Meyler et al., 2023), defining an appropriate “fatigue threshold” for designing and prescribing sessions using the W´_BAL-INT_ (Galán-Rioja et al., 2022)

Both the absolute and relative values of the HR followed a similar pattern to V˙O_2mean_ compared to the HR at CP_Test_. These acute cardiorespiratory responses of the HR associated with the steady state of V˙O_2mean_ during the work and recovery intervals below and slightly above CP, create a suitable oxygen steady state environment that promotes different physiological adaptations (Holloszy and Coyle, 1984). Furthermore, our results align with those found by Petit et al. (2007) regarding the estimation of V˙O_2_ derived from the HR. Although V˙O_2_ estimates derived from the HR have been reported to decrease at higher intensities (Pettitt et al., 2015), in our study V˙O_2_ and the HR followed a similar pattern to W´ balance even when performing repetitive intervals. In the present study, the HR showed a consistent steady state for intervals between 94 and 100% of HR_CP_ and between 86 and 92% of HR_max_. This indicates the sensitivity of the HR and the relative values of HR_max_ and HR_CP_. Therefore, the HR can be a good alternative during interval training, mainly because it follows similar patterns to those of V˙O_2_ and W' balance.

Regarding the blood lactate response during W´_BAL-INT_, similar responses were observed between the 3- and 7-min intervals (13.6 ± 3.3 and 12.9 ± 3.3 mmol·l^−1^, respectively). This can be attributed to the accumulation of metabolites (P_i_, [BLa̅], H^+^ and K^+^) in the severe intensity domain, where muscle metabolism and blood acid-base response do not stabilize above CP until reaching maximum (Burnley and Jones, 2018). Additionally, it was noted that the RPE during the first 2 min of both 30-s and 1-min intervals remained stable compared to the RPE during the CP_Test_, yet it increased from the 3-min work interval to its maximum at the end of the set. Similar responses were observed in a previous study (Okuno et al., 2011), where the RPE was highest from the middle to the end of the exercise duration.

This research study has several limitations that need to be acknowledged. The small sample size of 14 road cyclists, with predominance of males (13 males and 1 female), may impact the generalizability of the findings to larger and more diverse populations. Participants were classified as Tier 3 (national level) cyclists, which limits the diversity of skill levels and may not be fully representative for other skill levels and categories. Additionally, the utilization of specialized equipment, such as the Cyclus2 ergometer and the gas analyzer could limit the relevance of the findings to different exercise scenarios. The results may be specific to the study's setup, lab conditions, and exercise duration. Furthermore, there is a chance of measurement errors in variables such as V˙O_2_, HR, and [BLa̅] concentration, despite calibration efforts. Practical application might be challenging due to the controlled study nature. The conclusions might not directly apply to other sports or populations because the focus was on specific cyclists with unique characteristics.

## Practical Implications

In the current study, an intermittent work session utilizing the W´_BAL-INT_ model during work and recovery intervals performed above and below CP yielded a stable response in oxygen uptake (V˙O_2_), the heart rate (HR) and V˙O_2peak_, similar to that observed during the CP performance test. This type of intermittent work sessions could be effectively incorporated into the training regimen of master cyclists. It has the potential to enhance CP and maximal aerobic power, promoting physiological adaptations associated with maximal steady-state oxygen uptake and V˙O_2peak_. Furthermore, we found that the kinetics of energy balance (i.e., W´, fatigue) were responsive to interval intensity and accumulated time during training. A 4-min rest interval allowed for recovery up to a moderate level of fatigue (80–100% of W´ balance). These findings carry practical implications for understanding fatigue development and the exercise prescription across different intensity domains. They provide valuable insights for designing effective training programs that can benefit athletes, coaches, and physical trainers.

## Conclusions

This study provides evidence that the W´_BAL-INT_ training model in road master cyclists elicits steady-state physiological responses in mean oxygen kinetics, percent critical power, and the heart rate, comparable to those observed during the critical power performance test. However, different physiological responses were observed in peak oxygen kinetics, W´ energy balance and [BLa̅] concentration. These findings have important implications for understanding the development of fatigue in different intensity domains and can guide the prescription of intermittent training protocols.
